# The reactive metabolite target protein database (TPDB) – a web-accessible resource

**DOI:** 10.1186/1471-2105-8-95

**Published:** 2007-03-16

**Authors:** Robert P Hanzlik, Yakov M Koen, Bhargav Theertham, Yinghua Dong, Jianwen Fang

**Affiliations:** 1 Department of Medicinal Chemistry, University of Kansas, Lawrence, KS 66045, USA; 2 Bioinformatics Core Facility, University of Kansas, Lawrence, KS 66047, USA

## Abstract

**Background:**

The toxic effects of many simple organic compounds stem from their biotransformation to chemically reactive metabolites which bind covalently to cellular proteins. To understand the mechanisms of cytotoxic responses it may be important to know which proteins become adducted and whether some may be common targets of multiple toxins. The literature of this field is widely scattered but expanding rapidly, suggesting the need for a comprehensive, searchable database of reactive metabolite target proteins.

**Description:**

The Reactive Metabolite Target Protein Database (TPDB) is a comprehensive, curated, searchable, documented compilation of publicly available information on the protein targets of reactive metabolites of 18 well-studied chemicals and drugs of known toxicity. TPDB software enables i) string searches for author names and proteins names/synonyms, ii) more complex searches by selecting chemical compound, animal species, target tissue and protein names/synonyms from pull-down menus, and iii) commonality searches over multiple chemicals. Tabulated search results provide information, references and links to other databases.

**Conclusion:**

The TPDB is a unique on-line compilation of information on the covalent modification of cellular proteins by reactive metabolites of chemicals and drugs. Its comprehensiveness and searchability should facilitate the elucidation of mechanisms of reactive metabolite toxicity. The database is freely available at

## Background

The toxic effects of many simple organic chemicals, pollutants and even drugs are associated with their biotransformation to chemically reactive intermediates [1,2]. The latter in turn react covalently with cellular macromolecules, thus modifying their structure and potentially their function. Whereas modification of DNA can cause mutations or even cancer, protein modification is often associated with direct, acute cytotoxic effects. Protein covalent binding is usually detected by administering radioactive precursors to animals (or cell-based systems in vitro) and measuring the amount of radioactivity that becomes covalently attached to the macromolecules (i.e., not removable by dialysis, extraction or chromatography) [3].

The extent, time course and anatomical distribution of protein covalent binding generally correlate very well with similar measures of target organ toxicity in whole animals or cellular systems. However, a few prominent exceptions to this pattern are also known. For example, whereas bromobenzene and p-acetamidophenol (acetaminophen) are "textbook" pro-toxins, p-bromophenol [4] and m-acetamidophenol [5,6] are essentially nontoxic despite the fact that they undergo metabolic activation and covalent binding much like their toxic congeners. These and other examples indicate that while covalent binding is apparently *necessary *for toxicity, not all covalent binding is *sufficient *to cause toxicity. Since most biological responses to chemicals are highly structurally specific, to understand the mechanisms of cytotoxic responses it is imperative to understand the structural chemistry of protein covalent binding.

**Figure 1:**
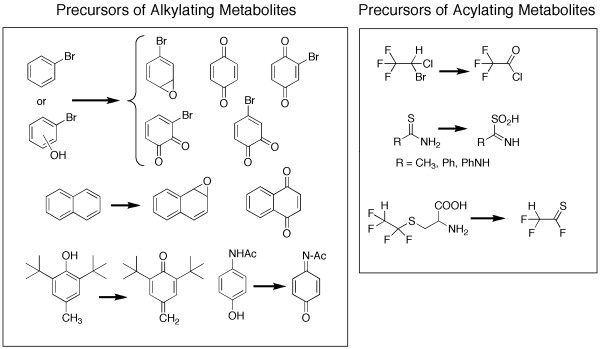
Formation of chemically reactive metabolites from stable pro-toxins.

The enzymes of xenobiotic metabolism generally have a rather broad substrate specificity that appears to be governed primarily by the functional group chemistry of potential substrates [7]. Since the early 1970s, considerable progress has been made in identifying the reactive metabolites formed from a large number of chemical functional groupings [8]. In the vast majority of cases they are electrophilic in nature. For example, epoxides, quinones and Michael acceptors generally react with cysteine sulfhydryl groups but also react with lysine, histidine and, to a lesser extent, methionine or even carboxylic acid side chains in proteins [9-11]. On the other hand metabolites such as acyl- and thioacyl halides and iminosulfinic acids show a strong tendency to acylate the epsilon-amino group of lysine side chains (Figure 1) [12-14].

In contrast to the wealth of structural information about cytotoxic metabolites, knowledge of their protein targets has been much slower to accumulate. The earliest identifications of reactive metabolite target proteins were based on classical isolation and N-terminal sequencing of radioactive proteins from animals treated with radiolabeled pro-toxins [15,16]. Subsequent reports have appeared sporadically and thus are scattered throughout the literature, making global comparisons or analyses difficult. Since the advent of modern mass spectrometry-based methods, longer lists of target proteins have been identified [17-20] but the literature still remains fragmented.

Our laboratory has been interested in exploring the possibility that different pro-toxins target a common subset of proteins whose covalent modification might be mechanistically significant to cytotoxicity [17,21,22]. To test this hypothesis required that we collect, organize and analyze essentially *all *of the publicly available information about well-identified target proteins that become adducted by reactive metabolites of organic chemical pro-toxins. The Reactive Metabolite Target Protein Database (TPDB) and the associated analysis software described herein were created to facilitate this task.

## Construction and content

The Reactive Metabolite Target Protein Database (TPDB) was implemented as an Oracle 9i relational database. An interactive web interface was created using Java Server Pages (JSP) and the Java Database Connectivity (JDBC) API was used to interface with the Oracle database. Currently our server runs on the LINUX operating system (RedHat AS3). The database is freely accessible on the web and the schema of the database design is available on the website.

## Populating the database

Computerized literature searches for "protein adducts" and similar terms yield more than 1500 references. However, the vast majority of these simply report occurrences of protein covalent binding detected in model systems, in occupational health screenings based on hemoglobin adducts ("molecular dosimetry"), or during in vitro screening tests associated with drug development pipelines. These reports generally fail to provide information about target protein identity or the relevance of the binding events to cytotoxicity and/or target organ toxicity. Thus, despite their value for kinetic and/or structural studies, reports of in vitro adduct formation in "model" systems using microsomes, purified enzymes, synthesized metabolites, or prepurified target proteins are not included in the TPDB at this time. Instead, we used information collected from original literature reports of specific identifications of target proteins for reactive metabolites derived from drugs and chemicals that have been well studied in the context of toxicology. Only results from studies conducted in vivo or in living cells/tissues are included, but subject to this restriction the collection is believed to be essentially complete. Most of the data came from experiments done in mice or rats, although a few human observations are included. Information about oxidative damage to proteins caused by reactive oxygen or -nitrogen species (ROS, RNS), or modifications by endogenous products of lipid peroxidation have not been included, although these areas are obvious opportunities for future expansion of the TPDB.

A simple but efficient data entry routine was implemented to facilitate populating the database. At each entry cycle the name of the small molecule (i.e., chemical or drug), the species and tissue involved, the name of the target protein identified, notes about the separation and identification methods employed, and the original literature reference were entered. In addition a flag (public/private) is set to allow us to enter, check and/or analyze "in house" and other data prior to incorporating it into the public database. For each entry a unique protein ID number was generated for internal use by the database software. When highly similar target proteins (e.g. *isoforms *from the same species or *orthologous *proteins from different species) were specifically and uniquely identified and reported as target proteins they were numbered and listed as distinct proteins in the database. However, for purposes of assessing commonality of targeting (see below), highly similar isoforms and orthologs are regarded as being the same protein according to a look-up table created for this purpose. A comments (text) field was also established for each protein entry. After the initial entry phase was complete, supplementary information was added for each protein as indicated in Table 1. Each entry was double-checked against the original literature prior to setting its flag to public access. Thus, we believe that the data contained in the TPDB are highly reliable.

**Table 1:** Information contained in the database for each protein entry

Protein name (and synonyms) found in the literature or other databases
Adduct-forming pro-toxin (small molecule)
Animal species and target tissue
Type of evidence on which protein identification was based
Separation method(s) used
Protein molecular weight and isoelectric point (MW and pI)
Swiss-Prot accession number (as a hyperlink)
NCBI accession number (as a hyperlink)
PDB entry (as a hyperlink, if available)
Full citation to original literature (with PMID number as a hyperlink)

## Searching the database

The web-accessible search page is organized into three sections plus a link to a Help Page that gives explanations and examples. The first section reports the results of four **automatic searches **to determine the number of small molecule pro-toxins and literature references in the database (currently 18 and 52, respectively). The software also reports the total number of protein hits (a hit is defined as one adduct on one protein reported in one reference) and the number of non-redundant protein entries in the database. Currently the TPDB contains 152 hits comprising 121 non-redundant proteins having 458 synonyms in the literature. This section also allows **string searches **for author surnames and protein names. For example, searching the string < dehyd > finds one "dehydratase" and 12 "dehydrogenase" proteins in the database. Clicking on any of the protein ID numbers generates an output table as described below.

The second section of the search page enables **custom searches **via four pull-down menus: Chemical, Target Species, Target Tissue and Protein Name. The default entry in each menu is "any (all);" thus the default search returns a table of all entries in the database (see Output section below). Custom searching is highly intuitive. For example, selecting just a chemical name (i.e. searching for < chemical, any, any, any >) will return all proteins in the database that are adducted by that chemical. This search can be refined by also selecting a target tissue. For example, searching < halothane, any, liver, any > will find all hepatic proteins known to be adducted by halothane metabolites. Numerous search combinations of < chemical, species, tissue, protein > are possible (although not all will produce results if there are no reported examples).

## Structure of information output

**Figure 2:**
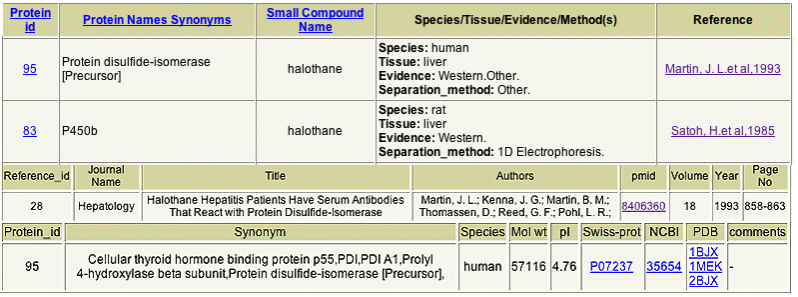
Examples of TPDB search output. Rows 1–3 show the header and two of nine lines from the output table generated by the search < halothane, any, any, any >. Rows 4 and 5 show the output result from the hyperlink "Martin, J. L. et al., 1983" in line 2. Rows 6 and 7 show the output result from the hyperlink "95" in line 2.

In general, search outputs are in the form of viewable, printable tables containing specific information and hyperlinks (Figure 2). For example, searching for target proteins of a small molecule (pro-toxin) produces a table containing the following information for each target protein of that small molecule: 1) the common name of the protein; 2) the small molecule name; 3) a summary of the target species and tissue, the type of evidence on which the identification was based, and the separation method(s) used; 4) the TPDB database protein ID# as a hyperlink to a secondary table that lists the ID#, names/synonyms, species of origin, molecular weight, isoelectric point, comments (if any were added when the protein was entered), Swiss-Prot and NCBI accession numbers (as active hyperlinks), and the PDB entry code (as a hyperlink, if available); and 5) a reference in the form of a hyperlink to the full citation information including the PubMed ID number (as an active hyperlink).

## Utility and discussion

In addition to the searches for specific items of information mentioned above, the TPDB also offers two types of **global searches**. The "Commonality Matrix" function produces a N × N matrix, where N is the number of small molecules in the database. Each diagonal element gives the number of proteins known to be adducted by the particular chemical whose row and column intersect on the diagonal. This allows a quick visual assessment of the extent to which various pro-toxins have been studied (and where additional studies might be needed). The off-diagonal elements give the number of proteins targeted by *both *of the chemicals whose row and column intersect at that off-diagonal element. Each element in the commonality matrix is also a hyperlink that leads to a table providing information about all the relevant proteins in the format described above.

Deeper insight into the common targeting of certain proteins by *multiple *pro-toxins is provided by a second global search function that provides a ranking of all proteins by the number of different chemicals which "hit" them. In this list, each protein ID number is a hyperlink that provides detailed information about that protein as described above. Using this function one finds that among the 121 non-redundant protein entries, only one protein (protein disulfide isomerase A1) is targeted by reactive metabolites of four different chemicals (acetaminophen, m-acetamidophenol, benzene and naphthalene). Only two proteins (selenium binding proteins 1 and 2) are targeted by reactive metabolites of three different pro-toxins, while 20 proteins are common to at least two of the 18 pro-toxins covered. The remaining 98 proteins in the database are all singletons as far as is known at present.

Thus, based on information collected from the open literature, commonality of protein targeting seems to be a pattern that is emerging slowly if at all. On the other hand, unpublished data from our laboratory reveals that bromobenzene (45 known target proteins in rat liver) [17,21,23] and thiobenzamide (71 known target proteins in rat liver; Koen et al., manuscript in preparation) share 28 protein targets in common. We expect that as target lists for individual compounds grow larger, commonality of targeting will start to become much more apparent. It is important to note, however, that covalent binding is a selective process and not a random process that is simply proportional to protein abundance, as discussed in ref. [24]. At present, the mechanistic significance of this commonality remains an open question, and a worthwhile direction for future research.

## Conclusion

The TPDB is a unique, web-accessible, searchable compilation of published data concerning the identification of cellular proteins targeted by electrophilic, chemically reactive metabolites of chemicals and drugs under in vivo or in-cell conditions. It is thought to be comprehensive or very nearly so in its listing of target proteins, and it is updated as new information emerges. Its data can be accessed and analyzed using a number of built-in search routines, and the results can be sorted and printed. The current content of 152 hits (121 non-redundant proteins) from studies with the 18 small molecule pro-toxins for which any protein target has been identified, is expected to rise significantly as recent advances in techniques for identifying proteins by mass spectrometry are more widely applied. It is hoped that diverse researchers and bio-informaticians, particularly those in industry [25], will both contribute to and analyze the content of the TPDB, and that as it grows it will prove useful for elucidating mechanisms of chemical toxicity.

## Availability and requirements

The database is freely accessible at  It has been tested to work with Mozilla Firefox 2.0 and Internet Explorer 6.0. Some features may not work with other browsers (e.g. Mac Safari 2.0).

## Authors' contributions

RPH conceived the project. YMK and RPH designedthe search capabilities and compiled the data. JF designed the relational database and its interface, and oversaw the IT operations of the project. BT, YD, and JF wrote the software for data entry, storage, searching and the web interface. RPH drafted the manuscript with input from YMK and JF. All authors have read and approved the final manuscript.
